# ANKRD17 induces pro-survival signaling pathways that enhance cellular invasion and migration during hepatocellular carcinoma tumorigenesis

**DOI:** 10.1016/j.isci.2025.112463

**Published:** 2025-04-17

**Authors:** Vincent W. Keng, Shan Su, Elyse S.T. Chui, Jeffrey C. To, Yao-jun Zhang, Xiao-Xiao Li

**Affiliations:** 1 The Hong Kong Polytechnic University Shenzhen Research Institute, Shenzhen 518057, China; 2 Department of Applied Biology and Chemical Technology, State Key Laboratory of Chemical Biology and Drug Discovery, The Hong Kong Polytechnic University, Hung Hom, Kowloon, Hong Kong SAR, China; 3 State Key Laboratory of Traditional Chinese Medicine Syndrome, The Second Affiliated Hospital of Guangzhou University of Chinese Medicine, Guangzhou 510405, China; 4 Department of Neurology, The Second Affiliated Hospital of Guangzhou University of Chinese Medicine, Guangzhou 510405, China; 5 Department of Neurology, Guangdong Provincial Hospital of Chinese Medicine, Guangzhou 510405, China; 6 Princess Margaret Cancer Centre, University Health Network, Toronto, ON, Canada; 7 Department of Liver Surgery, Sun Yat-sen University Cancer Center, Guangzhou 510060, China; 8 State Key Laboratory of Oncology in South China, Guangdong Provincial Clinical Research Center for Cancer, Sun Yat-sen University Cancer Center, Guangzhou 510069, China; 9 Research Center for Chinese Medicine Innovation, The Hong Kong Polytechnic University, Hung Hom, Kowloon, Hong Kong SAR, China

**Keywords:** cancer, cell biology, molecular biology

## Abstract

Metastasis is the primary cause of high mortality in patients with hepatocellular carcinoma (HCC) . A prior study identified ankyrin repeat domain 17 (*Ankrd17*) as a key gene linked to HCC metastasis. Through reverse genetics, it was observed that mouse liver tumors overexpressing *ANKRD17* exhibited a higher tumor load and increased expression of endothelial-mesenchymal transition (EMT) markers. Similarly, *ANKRD17* overexpression in human liver cell lines resulted in an amplified cellular motility and invasion capability, whereas knockdown studies reversed this effect. Abnormal regulation of signaling pathways was linked to increased metastasis and survival in cells overexpressing *ANKRD17*. Notably, the pro-metastatic discoidin domain receptor tyrosine kinase 1 (*DDR1*) gene was upregulated in these cells, and its suppression reduced motility and invasion without affecting AKT signaling. Clinically, higher *ANKRD17* expression correlated with aggressive HCC progression. These findings suggest that *ANKRD17* enhances metastatic progression in HCC by activating pro-metastatic and pro-survival pathways.

## Highlights

• *ANKRD17* induces signaling pathways that promote cellular invasion and migration
• *ANKRD17* induces the pro-metastatic *DDR1* gene
• Upregulation of *ANKRD17* and *DDR1* expression is commonly found in patients with advanced stage HCC

## Graphical abstract

**Figure:**
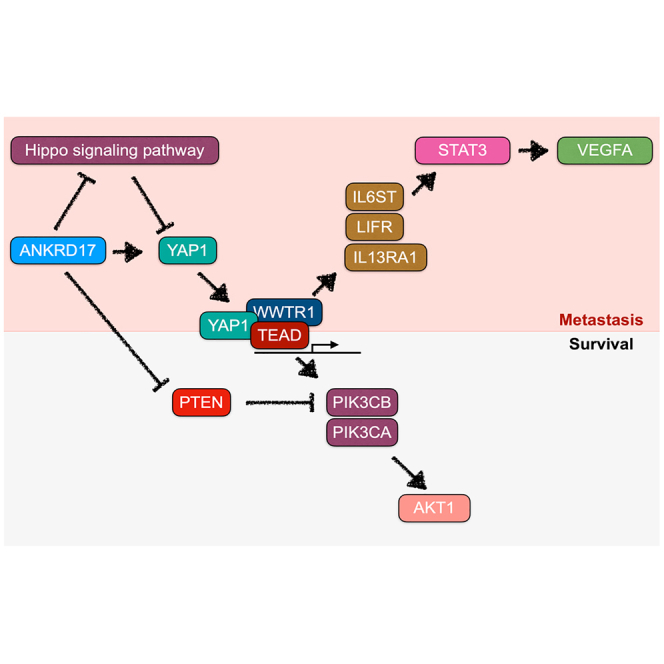

## Introduction

The development of hepatocellular carcinoma (HCC) is strongly associated with numerous risk factors, such as viral hepatitis infection, alcohol overconsumption, inflammation, hemochromatosis, obesity, diabetes, auto-immune liver disease, non-alcoholic fatty liver disease, and specific aspects of age and gender.^1^^,^^2^^,^^3^^,^^4^ Chronic liver ailments often result in liver inflammation leading to fibrosis, cirrhosis, and eventually, HCC.^5^ Severe cases may present tumors spreading from the original location to new areas. Tumor growth within the liver is termed as intrahepatic metastasis, while growth in other body organs is recognized as extrahepatic metastases. Primary sites for extrahepatic metastases are predominantly the lungs, followed by lymph nodes, bones, and adrenal glands.^6^^,^^7^^,^^8^ The metastasis process is a multi-faceted progression that initiates with local migration, invades the primary tumor site, and finally metastasizes to secondary areas.^9^^,^^10^^,^^11^

Metastasis significantly contributes to cancer-related deaths, causing nearly 90% of all such fatalities.^12^ This is largely due to the frequent incidence of metastasis in patients with HCC and the insufficient comprehension of its genetic underpinnings. According to the American Cancer Society’s 2023 report, metastasis is the leading cause of mortality among patients with HCC. The report also states that early localized HCC stage has a five-year survival rate of merely 36%, which drops drastically to 13% for regional spread stage and 3% for distant metastasis stage. HCC recurrence post liver resection and/or transplants is primarily due to intrahepatic spread, thus posing a significant challenge to HCC treatments.^13^^,^^14^^,^^15^ Therefore, it is crucial to gain a comprehensive understanding of the genetic mechanism(s) driving HCC-linked metastases to formulate more reliable therapeutic targets.

Ankyrin repeat domain 17 (*Ankrd17*), identified as a potential metastasis-associated gene, showed frequent mutagenic transposon integrations in the majority of lung metastases in our prior genetic search for HCC driver mutational genes.^16^ Ankrd17 belongs to the ankyrin repeat (ANK) family, featuring one of the most common functional motifs for protein-protein interaction.^17^ It is a transcriptional co-factor, localizes in the nucleus, and possesses two distinct clusters of 25 ankyrin repeats at its N-terminus, one nuclear exporting signal and one nuclear localization signal in the middle, and one RXL motif at its C-terminus.^18^^,^^19^ ANK-containing proteins have demonstrated association with transcriptional regulation, cell cycle progression, nuclear factor kappa B (NFKB) activation, immune pathways, and cytoskeleton modulation.^20^^,^^21^^,^^22^ Moreover, several studies have identified the deregulation of *Ankrd17* expression in various human cancers, including its role in bladder cancer metastasis.^23^^,^^24^^,^^25^^,^^26^ The Cancer Genome Atlas (TCGA) HCC database has documented significant overexpression of *ANKRD17* in primary tumor samples, with recorded patient survival rates correlating with expression levels. Notably, patients with HCC and metastases have also shown high expression levels of *ANKRD17*. Yet, its precise role and genetic mechanism(s) in the HCC-associated metastasis pathway remain unclear. In the present study, our findings highlight a crucial role of *ANKRD17* in promoting cellular migration and invasion by inducing several pro-metastatic and pro-survival signaling pathways, including the discoidin domain receptor tyrosine kinase 1 (*DDR1*) gene.

## Results

### Existing evidence points to genetic alterations in *ANKRD17* in patients with hepatocellular carcinoma and their potential implication in the process of liver metastasis

*ANKRD17* is altered in 7% (*n* = 25) of HCC patient samples (TCGA, *n* = 360), with a significant 64% of these patients presenting gene mutations such as high mRNA or gene amplification (Figure S1A). According to GEPIA,^27^ increased *ANKRD17* expression in patients with HCC severely diminishes the overall survival and disease-free survival rates (Figure S1B). Using UALCAN,^28^ *ANKRD17* expression has been found to be noticeably higher in primary tumors when compared to normal liver tissue (*p* = 1.56E-08). Moreover, it is also significantly linked to tumorigenesis in HCC grades 1 to 3 (Figure S1C). Notably, significantly elevated *ANKRD17* levels were found in patients with N1 HCC (metastases observed in 1–3 axillary lymph nodes, *n* = 4), when juxtaposed with patients with N0 HCC (*p* = 3.63E-02). This underscores the potential role of *ANKRD17* in the metastatic process (Figure S1D).

### *ANKRD17* overexpression enhances liver tumorigenesis, cellular migration, and invasive potential

**Figure 1:**
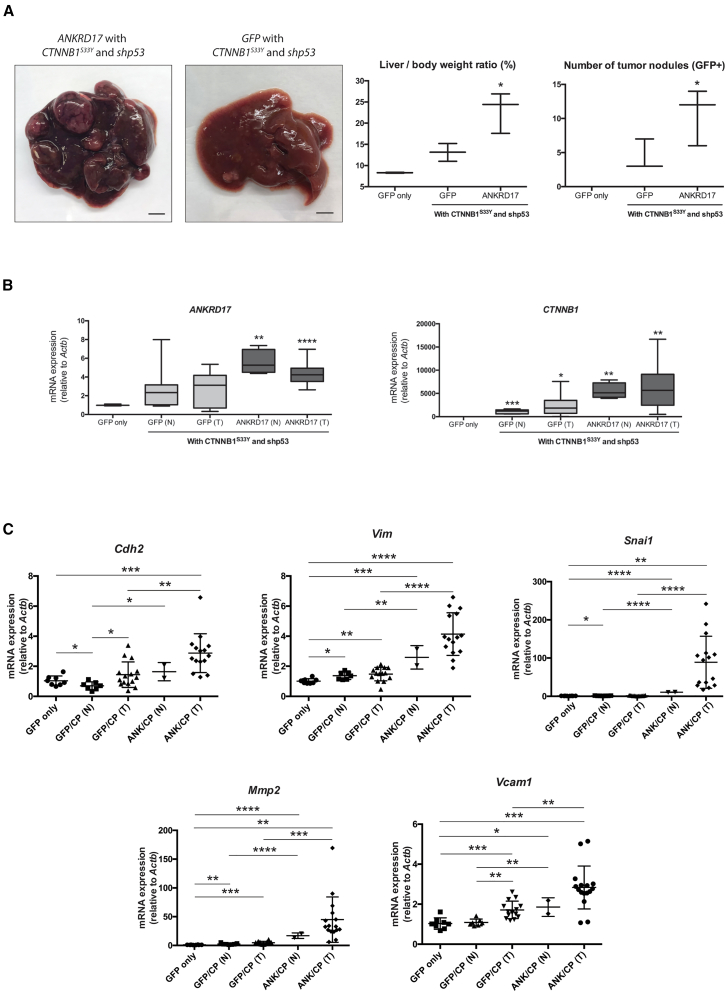
*ANKRD17* enhances tumorigenic and pro-metastatic characteristics during HCC disease progression (A) *ANKRD17*-overexpression in a *CTNNB1*^*S33Y*^ and *shp53* predisposed background (*n* = 3), resulted in larger liver to body weight percentage ratios and higher tumor burden in *Fah*/SB11 mouse injected livers at around 356-day PHI compared to control cohorts (GFP only, *n* = 2; GFP in a *CTNNB1*^*S33Y*^ and *shp53* predisposed background, *n* = 3). Scale bars, 0.5cm. (B) Quantitative PCR (qPCR) results showing upregulated *ANKRD17* and *CTNNB1*^*S33Y*^ expression levels in liver samples from experimental and control injected *Fah*/SB11 mice. (C) qPCR results show significant upregulation of EMT markers in liver tumors of experimental *Fah*/SB11 injected mice compared with control cohorts. Arbitrary value relative to *Actb* mRNA levels expressed as mean ± SD. GFP, *GFP* only control; GFP/CP, *GFP*, *CTNNB1*^*S33Y*^, and *shp53* control; ANK/CP, *ANKRD17*, *CTNNB1*^*S33Y*^, and *shp53* experimental; N, peripheral normal liver; T, tumor nodule. *p*, unpaired Student’s t test: ∗∗∗∗, *p* < 0.0001; ∗∗∗, *p* < 0.001; ∗∗, *p* < 0.01; ∗, *p* < 0.05.

The tumorigenic and metastatic potential of *ANKRD17* was confirmed using the *Fah*/SB11 transgenic mouse model. We implemented the *SB* transposon system to stably insert the *ANKRD17*-overexpressing vector into the hepatic genome of the *Fah*/SB11 transgenic mice. To simplify, the *ANKRD17*-overexpressing vector was co-injected with *CTNNB1*^*S33Y*^ and *shp53* plasmids into *Fah*/SB11 mice livers, utilizing the hydrodynamic tail vein injection technique (Figure S2A). The constitutive activation of *CTNNB1* and RNA interference against *Trp53* (*shp53*) were initiated to foment a predisposition to HCC, all the while analyzing the metastasis-inducing effects of *ANKRD17* overexpression. For comparative purposes, two historical control groups were used: *GFP* with *CTNNB1*^*S33Y*^ and *shp53*; and only *GFP*.^29^ At around 365-day post-hydrodynamic injection (PHI), mouse livers were isolated for downstream analyses. *ANKRD17*-overexpressing livers displayed larger liver to body weight ratios and increased tumor burden compared with control groups (Figure 1A). Approximately a year post-hydrodynamic injection (PHI), the mice’s livers were excised for further analysis. Livers which showed overexpression of *ANKRD17* revealed a more prominent liver to body weight ratio and heightened tumor burden compared to the control groups (Figure 1A). Successful overexpression was verified in injected mice livers through qPCR (Figure 1B). Though no metastasis was detected, notable upregulation of EMT markers, including *Cdh2*, *Vim*, *Snai1*, *Mmp2*, *Vcam1*, was observed in the tumors of *ANKRD17*-overexpressing mice livers by qPCR (Figure 1C).^30^^,^^31^^,^^32^^,^^33^ This suggests that the overexpression of *ANKRD17* in hepatocytes induced HCC tumorigenesis and increased its metastatic potential.

**Figure 2:**
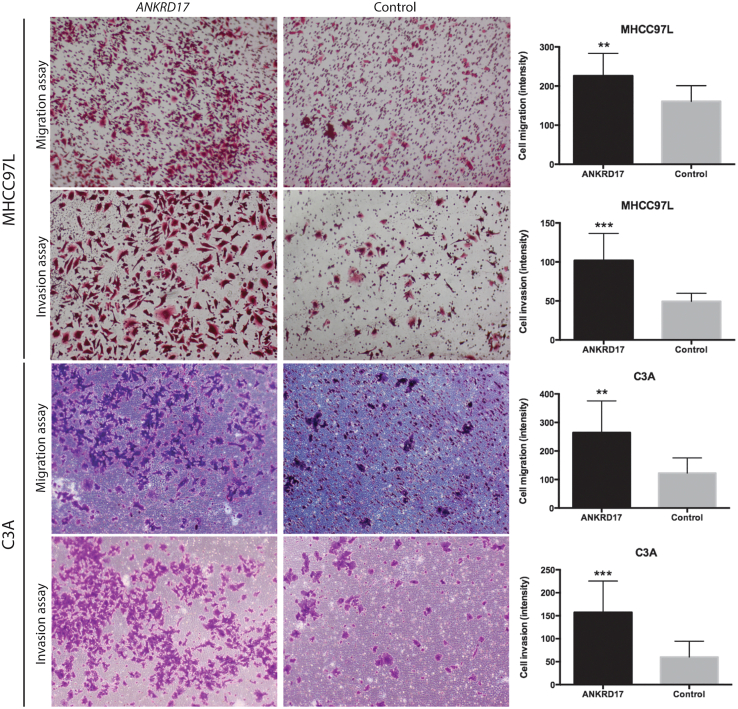
*ANKRD17*-overexpression enhanced cellular migration and invasion abilities in human liver cancer cell lines *ANKRD17*-overexpressing MHCC97L (top) and C3A (bottom) cells both exhibited significant increases in cellular motility and invasion abilities compared with control orange fluorescent protein (*OFP*)-transfected cells. Representative graphs of stained migrated or invaded cell intensity expressed as mean ± SD; *P*, unpaired Student’s t test: ∗∗∗, *p* < 0.001; ∗∗, *p* < 0.01.

To investigate the influence of *ANKRD17* on HCC-associated metastasis, we examined its effects on cell migration and invasion potential. This was achieved by utilizing the Transwell migration and Matrigel invasion chamber assays on two HCC cell lines, MHCC97L and C3A, selected due to their low-to-non-metastatic characteristics. Both cell lines exhibited an increase in cell mobility and invasion capabilities when transfected with the *PB* transposon *ANKRD17*-overexpressing vector, compared with cells transfected with the control *OFP*-overexpressing vector (Figure 2). No significant alterations were detected in the cell proliferation rates between the *ANKRD17*-overexpressed cells and the control cells, as established by an MTS assay performed on both cell lines (Figure S3A).

### *ANKRD17* induces pro-metastatic and pro-survival activity by dysregulating several important signaling pathways

To investigate the molecular mechanisms underlying the enhanced migratory and invasive capabilities induced by *ANKRD17*, RNA-sequencing (RNA-seq) was conducted on total RNA extracted from *ANKRD17*-overexpressing and control *OFP*-overexpressing MHCC97L cells. Upon examination, 259 DEGs were identified, with 221 genes being upregulated and 38 genes downregulated.

These DEGs were further analyzed via Ingenuity Pathway Analysis (IPA), revealing that the STAT3 (-log *p*-value = 1.45), tumor microenvironment (-log *p*-value = 0.684), and IL-6 signaling pathways (-log *p*-value = 0.254), often associated with HCC tumorigenesis and metastasis, were notably enriched among the upregulated DEGs (Table S1). In contrast, the HIPPO (-log *p*-value = 2.2) and PTEN (-log *p*-value = 0) signaling pathways, also associated with HCC tumorigenesis and metastasis, were enriched among the downregulated DEGs (Table S2).

**Figure 3:**
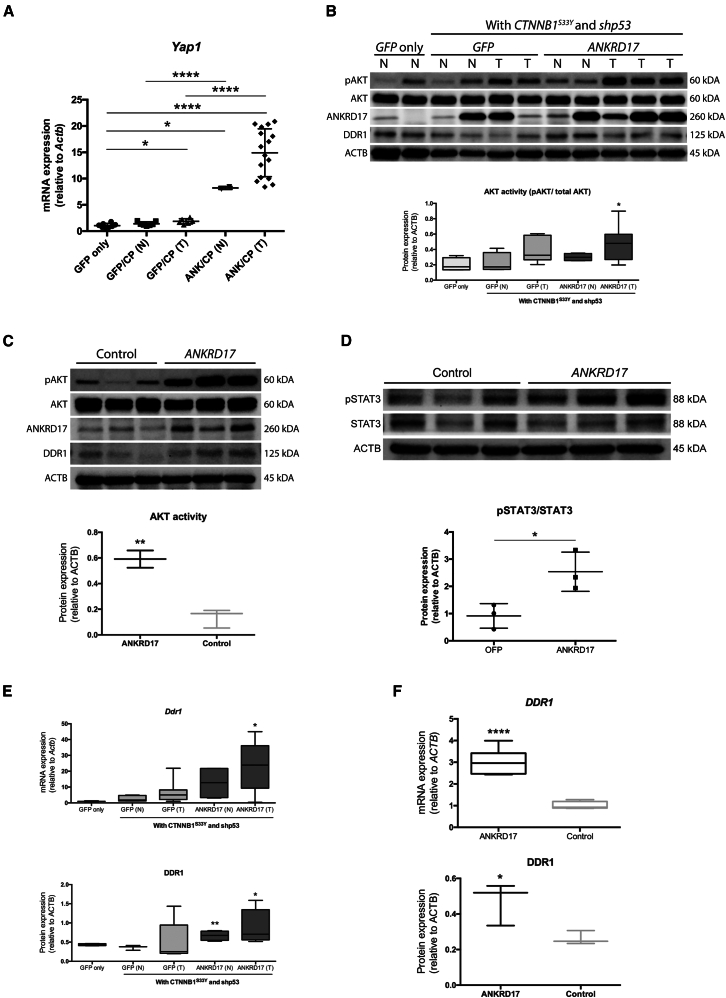
*ANKRD17*-overexpression induces the dysregulation of several important signaling pathways (A) Liver tumors from experimental mice demonstrating dysregulated HIPPO signaling pathway with significantly higher expression of *Yap1* than control cohorts. Quantitative PCR results of *Yap1* relative to *Actb* levels expressed as mean ± SD. GFP, *GFP* only control; GFP/CP, *GFP*, *CTNNB1*^*S33Y*^, and *shp53* control; ANK/CP, *ANKRD17*, *CTNNB1*^*S33Y*^, and *shp53* experimental; N, peripheral normal liver; T, tumor nodule. (B) Representative Western blot demonstrating dysregulated AKT/PTEN signaling pathway in tumors (T) and their peripheral normal (N) tissues. Semi-quantitative analyses of AKT activity (pAKT/total AKT) relative to ACTB levels expressed as mean ± SD. (C) Representative Western blot demonstrating dysregulated AKT/PTEN signaling pathway in *ANKRD17*-overexpressing MHCC97L cells. Semi-quantitative analyses of AKT activity (pAKT/total AKT) relative to ACTB levels expressed as mean ± SD. (D) Representative Western blot demonstrating dysregulated STAT3 signaling pathway in *ANKRD17*-overexpressing MHCC97L cells. Control, *OFP*-overexpressing cells. Semi-quantitative analyses of STAT3 activity (pSTAT3/total STAT3) relative to ACTB levels expressed as mean ± SD. (E) Liver tumors from experimental mice demonstrated significantly higher expression of *Ddr1* than control cohorts at both the transcriptional (top) and translational (bottom) levels. Western blot for DDR1 as shown in (B). Semi-quantitative analyses of *Ddr1* and DDR1, relative to *Actb* and ACTB levels, respectively, expressed as mean ± SD. (F) Induced expression of *DDR1* at both the transcriptional (top) and translational (bottom) levels in *ANKRD17*-overexpressing MHCC97L cells. Western blot for DDR1 as shown in (C). Semi-quantitative analyses of *DDR1* expression at both the transcriptional (top) and translational (bottom) levels relative to *ACTB* and ACTB levels, respectively, expressed as mean ± SD. *P*, unpaired Student’s t test: ∗∗∗∗, *p* < 0.0001; ∗∗∗, *p* < 0.001; ∗∗, *p* < 0.01; ∗, *p* < 0.05.

*ANKRD17*-overexpressing cells and tumor samples revealed significantly higher expression of yes-associated protein 1 (*Yap1*), indicative of a dysregulated HIPPO signaling pathway (Figure 3A), and notably elevated AKT phosphorylation at Ser473, an indicator of a dysregulated PTEN signaling pathway (Figures 3B and 3C, respectively). This suggests that *ANKRD17* promotes the pro-metastatic transcription of *Yap1* and pro-survival AKT phosphorylation during HCC metastatic progression by dysregulating the HIPPO and PTEN signaling pathways, respectively. The STAT3 signaling pathway also appeared to be activated in *ANKRD17*-overexpressing MHCC97L cells. Phosphorylated STAT3 levels were significantly higher in *ANKRD17*-overexpressing than in *OFP*-overexpressing control MHCC97L cells (Figure 3D). Consistently, *IL6R* and *STAT3* were also upregulated in *ANKRD17*-overexpressing C3A cells when compared with *OFP*-overexpressing control (Figure S3B).

In a noteworthy observation, *DDR1*, a gene previously reported to be involved with metastasis in various types of cancer, was discovered to be significantly upregulated.^34^^,^^35^^,^^36^^,^^37^^,^^38^^,^^39^ Both liver tumors and HCC cells overexpressing *ANKRD17* showed significant upregulation of *DDR1*, suggesting that ANKRD17 triggers the pro-metastatic transcription of DDR1 during HCC metastasis (Figures 3E and 3F, respectively). The upregulation of DDR1 was also confirmed by IHC staining in overexpressing *ANKRD17* livers of *Fah*/SB11 mice (Figure S3C).

**Figure 4:**
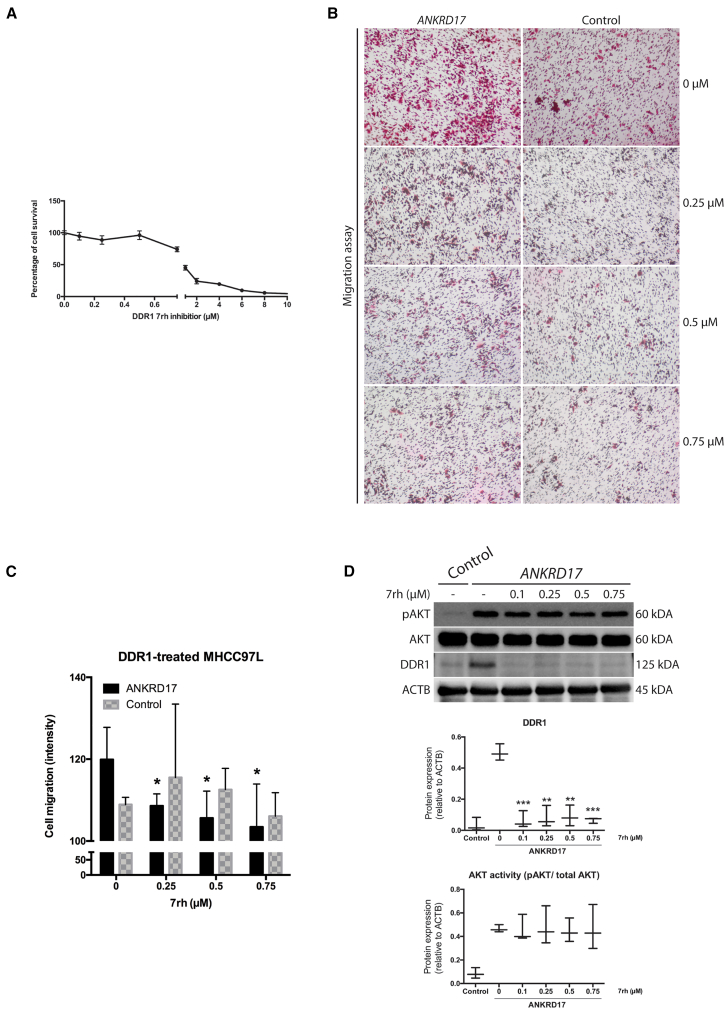
Inhibition of *DDR1* suppressed *ANKRD17*-induced cellular migration ability in HCC cancer cell line (A) Cell survival assay of DDR1 inhibitor 7rh at different concentrations to determine the sub-cytotoxic conditions for subsequent experiments. (B) Cell motility assessed by transwell migration assay using DDR1 inhibitor 7rh at 0, 0.25, 0.5, and 0.75 μM in *ANKRD17*-overexpressing MHCC97L cells. (C) Semi-quantitative analyses of the cellular migration results from (B) expressed as mean ± SD. (D) Representative Western blot demonstrates reduction in DDR1 levels but no alterations in AKT phosphorylation at Ser473 were detected with DDR1 inhibitor 7rh at all tested concentrations. Control, *OFP*-overexpressing cells. Semi-quantitative analyses of DDR1 and AKT activity (pAKT/total AKT) relative to ACTB levels expressed as mean ± SD. *P*, unpaired Student’s *t test*: ∗∗∗, *p* < 0.001; ∗∗, *p* < 0.01; ∗, *p* < 0.05.

To confirm this hypothesis, *ANKRD17*-overexpressing cells were treated with the DDR1 inhibitor 7rh. Cells were first treated with various concentrations of DDR1 inhibitor 7rh for 48 h, followed by MTS assay to determine the sub-cytotoxic concentrations for use in subsequent experiments (Figure 4A). After 48 h of treatment with different concentrations of DDR1 inhibitor 7rh (≤0.75 μM), a reduced cell migration phenotype was observed in the *ANKRD17*-overexpressing cells, suggesting a crucial role of DDR1 in ANKRD17-induced cell migration (Figures 4B and 4C). Interestingly, no changes in AKT signaling activity were observed as a result of DDR1 inhibition, indicating that this pathway depends on ANKRD17 rather than DDR1 activation (Figure 4D).

Additionally, RNA interference assay targeting *ANKRD17* was conducted *in vitro* to confirm its role in metastasis. The SNU449 cell line was chosen due to its high level of *ANKRD17* expression (The Human Protein Atlas website). Short hairpin RNAs (shRNAs) targeting *ANKRD17* were successfully transfected into SNU449 cells and exhibited resistance to puromycin (*data not shown*). Successful knockdown of *ANKRD17*, averaging 40%, was achieved with the gene-specific shRNAs, as demonstrated by semi-quantitative PCR analyses (Figures S4A–S4C). Wound healing assay performed on two *ANKRD17*-knockdown clones (C and D) demonstrated statistically significant delay in wound healing when compared with the scramble control (Figures S4D and S4E).

### Elevated *ANKRD17* and *DDR1* expression levels in patients with advanced stage HCC

Online clinical GEO DataSets (GSE6764 and GSE40367) were utilized to assess the implications of ANKRD17 and DDR1 in patients with HCC.^40^^,^^41^^,^^42^ Findings from the GSE6764 database revealed a common overexpression of both *ANKRD17* and *DDR1* in advanced stage HCC cases, particularly in patients with cancer that has spread to lymph nodes or other organs (Figure S5A). Additionally, the GSE40367 database indicated that *ANKRD17* was significantly overexpressed in lung metastases compared to primary tumors (Figure S5A). Moreover, *DDR1* expression was relatively higher in lung metastases compared to their primary tumors. These observations collectively imply that *ANKRD17* and *DDR1* are significantly implicated in patients with HCC-associated metastasis.

In corroboration with our results, both ANKRD17 and DDR1 were detected in 5 lung metastasis samples taken from patients with HCC (*n* = 9) (Table S3 and Figure S6).

### Expression levels of *ANKRD17* and affected signaling pathways in orthotopic transplanted human HCCLM3-RFP cell line

The GEO DataSet GSE94016, which mimics tumor growth and metastasis in patient’s liver, was employed to assess the role of *ANKRD17* and its impact on signaling pathways.^43^ Within the GSE94016 database, there was a significant increase in *ANKRD17* expression detected after a 4-week period of orthotopic implantation (Figure S5B). In line with our findings, a downregulation of the HIPPO and PTEN signaling pathways was also observed, indicated by a considerable escalation in *YAP1* and *PIK3CA* expression, respectively (Figure S5B). Additionally, *PTEN* itself was also significantly downregulated after a 4-week period of orthotopic implantation (Figure S5B). There was an evident upregulation of the IL6 and STAT3 signaling pathways, marked by notable increases in *IL6ST* and *LIFR*, along with *STAT3* and *VEGFA* expression levels, respectively (Figure S5C).

## Discussion

A preceding *in vivo* study utilizing *SB* insertional mutagenesis screening to discern driver genes responsible for hepatocellular carcinoma (HCC) generated a phenotype mirroring the human disease, including metastasis. Intriguingly, mutagenic transposition at the *Ankrd17* gene locus, found exclusively in lung metastases, solicited our exploration of this gene in HCC-associated cancer spread.^16^ ANKRD17 serves as a transcriptional co-factor and, due to its unique structural property, it has been posited to function in nucleo-cytoplasmic transport.^18^^,^^19^ However, the functional role of *ANKRD17* in HCC tumorigenesis and metastasis is yet to be entirely understood. Based on our *SB* insertional mutagenesis screening, we hypothesize that *ANKRD17* may regulate the metastatic process in HCC tumorigenesis. We used both *in vitro* and *in vivo* experiments to test this hypothesis and establish a connection between *ANKRD17* and cancer aggressiveness.

Our current study shows that ANKRD17 could trigger traits of HCC transformation in the liver and promote metastatic activity (Figure 1). Also, its overexpression in human HCC cell lines amplified both cellular motility and invasion capabilities (Figure 2). Consistent with the RNA-seq data and bioinformatics analyses, *ANKRD17*-overexpression repressed HIPPO and PTEN signaling pathways, while increasing the IL6/STAT3 signaling pathway (Figure 3). Our data indicates that *ANKRD17* propels cellular migration and invasion in HCC progression by modulating these crucial pro-metastatic and pro-survival signaling pathways. These findings are supported by research indicating its nuclear import and cofactor properties toward YAP, which instigates target gene transcriptions promoting cancer’s proliferation, survival, stem cell maintenance, and metastasis.^18^^,^^19^^,^^23^ Its dysregulation in various human cancers and its role in bladder cancer-related metastasis have also been demonstrated.^23^^,^^24^^,^^25^^,^^26^ RNA interference assay targeting *ANKRD17* conducted in SNU449 cells also confirmed its role in metastasis. Wound healing assay performed on *ANKRD17*-knockdown clones demonstrated statistically significant delay in wound healing when compared with scramble control (Figures S4D and S4E). Interestingly, other studies have shown that knockdown of ANKRD17 inhibits DNA replication and blocks cell cycle progression.^20^ In addition, double siRNA against ANKHD1 and ANKRD17 causes apoptosis in mammalian cell lines.^19^ Additionally, our findings suggest that *ANKRD17* may activate other pro-metastatic and pro-survival pathways such as the PTEN/PI3K/AKT signaling, which manages multiple cellular activities comprising survival and cell migration, as well as the STAT3 signaling vital for the many intricate steps involved in metastasis.^44^^,^^45^ Consistent with our results, HIPPO, PTEN, and STAT3 signaling pathways were also dysregulated after 4-week orthotopic implantation of human HCCLM3 cell line in GEO DataSet GSE94016 (Figure S5). Based on our RNA-seq data, *DDR1* was one of the top upregulated genes in *ANKRD17*-overexpressing cells. *ANKRD17*-overexpression consistently induced *DDR1* upregulation in both *in vitro* and *in vivo* experiments (Figure 3). *DDR1* is a transmembrane tyrosine kinase fibrillar collagen receptor responsible in cell attachment, migration, survival, and cell growth.^46^ *DDR1* has been extensively studied for its tumorigenic and metastatic characteristics in human cancers, including HCC.^47^^,^^48^^,^^49^^,^^50^ Its overexpression has been well-documented in many cancers, such as non-small cell lung carcinoma, metastatic breast cancer, and aggressive neuroendocrine prostate cancer.^24^^,^^37^^,^^38^ Other studies have shown that *DDR1* can promote matrix metalloproteinases (MMPs) secretion to induce extracellular matrix (ECM) degradation and tumor metastasis, while its inhibition has been reported to hamper metastatic colonization.^38^^,^^51^^,^^52^^,^^53^^,^^54^^,^^55^ DDR1 signaling can influence the expression of collagen genes, contributing to extracellular matrix organization and regulate the expression of E-cadherin, which is important in maintaining epithelial cell integrity.^46^^,^^56^ DDR1 can also influence the expression of genes involved in cell survival and apoptosis.^57^ DDR1 signaling can also modulate the expression of growth factors such as VEGF, impacting angiogenesis.^58^ To understand the underlying relationship of the changes of DDR1 expression in *ANKRD17*-induced cellular motility and invasion, *in vitro* DDR1 inhibition was performed. The role of *DDR1* in *ANKRD17*-induced cellular motility and invasion was confirmed using *in vitro* DDR inhibition assays, which reversed the metastatic phenotype in *ANKRD17*-overexpressing cells (Figure 4). However, no alterations in AKT phosphorylation were observed, suggesting that the pro-survival AKT pathway is the direct target of *ANKRD17* instead of *DDR1*. These data suggest *DDR1* may be the potential key component of *ANKRD17*-related metastasis in HCC. In addition, good correlation between ANKRD17 and DDR1 was also observed in ∼56% of lung metastasis samples taken from patients with HCC (Figure S6). *DDR1* was significantly elevated in advanced HCC patient samples, while *ANKRD17*-overexpression was also reported in advanced stage HCC samples and, importantly, in lung metastases compared to primary liver tumors (Figure S5).

Our current study provides pioneering mechanistic evidence that *ANKRD17* contributes to cellular migration and invasion during HCC progression by modulating several critical signaling pathways. In particular, the pro-metastatic *DDR1*, consistently upregulated during *ANKRD17*-mediated cell migration and invasion, and whose inhibition reversed this phenotype, emerges as a crucial mediator in *ANKRD17*-induced cellular migration and invasion. Therefore, our findings enrich the understanding of ANKRD17’s role in HCC-related metastasis, suggesting it as a new therapeutic target for curtailing HCC-induced metastasis and disease recurrence.

### Limitations of the study

The mechanistic role of *ANKRD17* in driving the metastatic potential of HCC may vary across different molecular subclasses of the disease. This variability suggests that *ANKRD17* could interact with distinct molecular pathways or cellular processes depending on the specific subclass of HCC. However, the current study did not fully explore these subclass-specific mechanisms. Further research is needed to comprehensively understand how *ANKRD17* contributes to metastasis in each molecular subclass of HCC, which could potentially lead to more targeted therapeutic strategies.

## Resource availability

### Lead contact

Further information and requests for resources and reagents should be contacted directly to and will be fulfilled by the lead contact, Vincent W. Keng (vincent.keng@polyu.edu.hk).

### Material availability

This study did not generate new unique reagents.

### Data code availability

• Data reported in this article will be shared by the lead contact upon request.
• This article does not report original code.
• Any additional information required to reanalyze the data reported in this article is available from the lead contact upon request.

## Acknowledgments

We would like to thank the Centralised Animal Facility (CAF), The Hong Kong Polytechnic University, for their excellent technical assistance with experimental animals described in this study. We would like to thank Dr Lilian H. Lo for her technical assistance in performing the validation experiments.

V.W.K. was supported by Project 82073134 of the 10.13039/501100001809National Natural Science Foundation of China; State Key Laboratory of Chemical Biology and Drug Discovery (1-BBX8); The Hong Kong Polytechnic University Research Center for Chinese Medicine Innovation (1-BBCT); 10.13039/501100004377The Hong Kong Polytechnic University/UGC internal funding (1-ZVST, 1-ZVY7, 1-WZ52 and 1-WZAJ). X.X.L was supported by The Hong Kong Polytechnic University RAP Start-up Foundation (I2021A016).

## Author contributions

Vincent W. Keng: Conceptualization, methodology, validation, formal analysis, investigation, writing – original draft, supervision, project administration, funding acquisition. Shan Su: validation, formal analysis, investigation, writing – review and editing, and visualization. Elyse S.T. Chui: validation, formal analysis, investigation, writing – review and editing, and visualization. Jeffrey C. To: validation, investigation, and writing – review and editing. Yao-jun Zhang: resources, data curation, and writing – review and editing. Xiao-Xiao Li: methodology, resources, writing – review and editing, supervision, project administration, and funding acquisition.

## Declaration of interests

The other authors declare no conflict of interest.

## STAR★Methods

### Key resources table

**Table:** 

REAGENT or RESOURCE	SOURCE	IDENTIFIER
**Antibodies**		

ACTB	Cell Signaling Technologies	#3700; RRID: AB_2242334
ANKRD17	Abcam	ab85726; RRID: AB_1860952
ANKRD17	Novus Biologicals	NB100-86996; RRID: AB_2227388
DDR1	Cell Signaling Technologies	#5583; RRID: AB_10694842
Phospho-DDR1 (Tyr513)	Thermo Fisher Scientific	PA5-104588; RRID: AB_2816063
Phospho-AKT (Ser473)	Cell Signaling Technologies	#4060; RRID: AB_2315049
Total AKT	Cell Signaling Technologies	#4691; RRID: AB_915783
STAT3	Abcam	ab68153; RRID: AB_2889877
Phospho-STAT3	Abcam	ab76315; RRID: AB_1658549

**Biological samples**		

Metastatic Lung Samples	Sun Yat-sen University Cancer Center	B2020-350

**Chemicals, peptides, and recombinant proteins**		

NTBC	Sobi	800000
RNAlater Stabilization Solution	Thermo Fisher Scientific	AM7020
TRIzol Reagent	Thermo Fisher Scientific	15596026
DDR1 Inhibitor 7rh	Sigma-Aldrich	SML1832
Puromycin	Thermo Fisher Scientific	A1113803

**Critical commercial assays**		

Qproteome Mammalian Protein Prep Kit	Qiagen	37901
Gateway LR Clonase II Enzyme Mix	Thermo Fisher Scientific	11791020
ViaFect Transfection Reagent	Promega	E4981
PrimeScript RT Master Mix	Takara	RR036B
SYBR Green Master Mix	Takara	RR420A
Millicell Hanging Cell Culture Insert	Merk	MERK-00001
Matrigel Invasion Chamber	Corning	354480
CellTiter 96® AQ_ueous_ One Solution Cell Proliferation Assay	Promega	G3582
ANKRD17 Human shRNA Plasmid Kit	Origene	TL306698

**Experimental models: Cell lines**		

MHCC97L	Prof. Terence K. Lee	The Hong Kong Polytechnic University
C3A	ATCC	TCP-1011
SNU449	ATCC	TCP-1011

**Experimental models: Organisms/strains**		

Fah/SB11 Mice (C57BL/6J X 129/Sv)	N/A	N/A

**Recombinant DNA**		

ANKRD17 cDNA	GeneCopoeia	Z8073
pENTR11 Dual Selection Vector	Thermo Fisher Scientific	A10467
pT2/GD-DEST-EGFP	To et al.^59^	N/A
pPB/SB-GFP-Puro	To et al.^59^	N/A

**Software and algorithms**		

ImageJ	NIH	Version 1.40J
Prism Software	GraphPad	Version 10.4.1

### Experimental model and study participant details

#### Transgenic mouse model

All animals were housed in the Centralised Animal Facility (CAF) at The Hong Kong Polytechnic University (HKPU) in Hong Kong SAR, China. The humane care of the animals was prioritized, and ethical approval was sought from the Animal Subjects Ethic Sub-Committee (ASESC 12/05), a research unit in line with CAF at HKPU. The study was conducted on fumarylacetoacetate hydrolase (*Fah*)-deficient mice, with a mixed genetic background of C57BL/6J X 129/Sv, which carry the ubiquitously expressed Sleeping Beauty (*SB*) transposase transgene that has been inserted into the *Rosa26* locus. These mice, known as *Fah*/SB11 mice,^16^^,^^29^^,^^60^^,^^61^ were maintained on drinking water supplemented with nitisinone (NTBC, Sobi) at a final concentration of 6 μg/mL and normal chow *ad libitum.*^62^

#### Transposon expression vectors and hydrodynamic tail vein injection

In our experimental procedures, we administered transposon expression vectors (20 μg each vector) into the livers of 45-day old *Fah*/SB11 male mice via hydrodynamic tail vein injection.^59^^,^^60^^,^^61^^,^^63^^,^^64^^,^^65^ The *ANKRD17* cDNA (NM_198889.2), sourced from GeneCopoeia, was cloned into the pENTR11 Dual Selection Vector (Thermo Fisher Scientific) to create pENTR-ANKRD17. This product was then integrated into the *SB* gene delivery destination vector pT2/GD-DEST-EGFP using the Gateway LR Clonase II Enzyme Mix (Thermo Fisher Scientific) reaction, leading to the creation of pT2-GD-ANKRD17-EGFP.^59^ Our experimental cohort were injected with *ANKRD17*, constitutively active catenin beta 1 (*CTNNB1*^*S33Y*^), and shRNA against transformation related protein 53 (*Trp53* - *shp53*) transposon vectors. Conversely, our control cohorts received injections of either green fluorescent protein (*GFP*), *CTNNB1*^*S33Y*^ and *shp53* transposon vectors, or simply the *GFP* transposon vector.^29^ Post-injection, NTBC-supplemented water was promptly switched with regular drinking water and normal chow *ad libitum* (Figure S2A). After 365-day post-hydrodynamic injection, the mice were euthanized, and their livers were harvested for further analysis.

#### Liver tumor analyses

The mouse’s entire liver was firstly harvested, and its weight was measured after being rinsed with cold phosphate-buffered saline (PBS). We then identified, counted, and isolated the tumor nodules, dividing them specifically for RNA and protein evaluations, and histological examinations. Samples set aside for RNA extraction were securely stored in an RNAlater Stabilization Solution (Thermo Fisher Scientific), at a temperature of −80°C. The extraction process was executed using Trizol Reagent (Thermo Fisher Scientific), strictly in accordance with the manufacturer’s protocol. Protein extraction was similarly conducted using the Qproteome Mammalian Protein Prep Kit (Qiagen), guided by the manufacturer’s protocol.

#### Cell culture and transfection

The human HCC cell lines MHCC97L and C3A (HepG2/C3A, derivative of HepG2) were cultured in Dulbecco’s Modified Eagle’s Medium, while SNU449 was cultured in RPMI1640 Medium. All culture media were supplemented with 10% fetal bovine serum and 1% antibiotic-antimycotic, and cells were maintained in a humidified 5% CO_2_ incubator at 37°C. The MHCC97L cell line was generously donated by Professor Terence K. Lee from HKPU, while the C3A and SNU449 cell lines were purchased from ATCC. All cell culture media and reagents were sourced from Gibco (Thermo Fisher Scientific).

Our study employed the piggyBac (*PB*) transposon system to stably integrate and overexpress the *ANKRD17*-overexpression vector in human HCC cell lines. In brief, *ANKRD17* cDNA was cloned into the *PB* transposon expression vector using the Gateway LR Clonase II Enzyme Mix (Thermo Fisher Scientific), resulting in the creation of pPB/SB-ANKRD17-GFP-Puro.^59^ A control expression vector was also constructed, consisting of the orange fluorescent protein (*OFP*), to yield pPB/SB-OFP-Puro via a similar methodology. Subsequently, pPB/SB-ANKRD17-GFP-Puro or pPB/SB-OFP-Puro was co-transfected with the *PB* transposase vector into human HCC cell lines using the ViaFect Transfection Reagent (Promega), adhering to the manufacturer’s instructions throughout the process. Post-transfection selection process using puromycin (Thermo Fisher Scientific) was carried out to enrich for stably transfected cells (Figure S2B).

#### Patients and specimens

Metastatic lung samples were obtained from 9 patients of Han Chinese ethnicity with pathologically confirmed HCC who underwent resection at the Sun Yat-sen University Cancer Center (Table S3). The study protocol was approved by the Institutional Review Board of Sun Yat-sen University with the ethical approval number B2020-350. This study utilized anonymized clinical data collected as part of routine patient care. In accordance with the ethical guidelines of our institution and national regulations, written informed consent was waived by the ethics committee.

### Method details

#### qPCR analyses

We utilized total RNA from HCC cells and tissue samples as a template for cDNA synthesis, employing the PrimeScript RT Master Mix (Takara). The diluted cDNA was then amalgamated with SYBR Green Master Mix (Takara) and a set of targeted primers in line with the manufacturer’s protocol. The expression of target mRNA was then ascertained using the QuantStudio 7 Flex Real-Time PCR System (Thermo Fisher Scientific), facilitated by the University Research Facility in Life Sciences at HKPU. The sequences for the targeted gene primers are provided in Table S4.

#### Transwell migration and matrigel invasion chamber assays

We procured transwell migration inserts from Millicell and Matrigel invasion chambers from Merck and Corning, respectively. The human liver cancer cell lines, consisting of 5 x 10^4^ cells each, were placed in a serum-free medium in the upper chamber. Simultaneously, serum-containing medium was introduced into the lower chamber, following the manufacturer’s guidelines. Post 48 h of incubation, the cells that had either migrated or invaded into the lower chamber were fixed using a 4% paraformaldehyde solution in PBS and subsequently stained with hematoxylin. We used 100X magnification to capture images of the migrated cells, which were then analyzed and counted using ImageJ.

#### Cell proliferation and cytotoxicity assays

Both cell proliferation and cell viability were evaluated using the CellTiter 96 AQ_ueous_ One Solution Cell Proliferation Assay (MTS) (Promega), following the manufacturer’s suggested protocols. To gauge the rate of cell proliferation, 1 x 10^3^ cells were seeded onto 96-well plates. Following overnight growth, the cells were exposed to the MTS solution, then incubated for 1.5 h before measuring the absorbance at 490nm using a microplate reader (Labexim Products). Cell viability was tested daily, for a duration of five days, beginning from the day the cells were seeded. For the cytotoxicity experiments, the cells were exposed to DDR1 inhibitor 7rh (Sigma-Aldrich) at varying concentrations (0, 0.1, 0.25, 0.5, 0.75, 1, 2, 4, 6, 8, 10 μM) over a span of 48 h. Following this, the MTS assay was performed in the same manner as aforementioned.

#### ANKRD17 RNA interference

A set of short hairpin RNA (shRNA) constructs, including four gene-specific shRNA expression vectors targeting *ANKRD17* and a scrambled negative control, was purchased from Origene. The four *ANKRD17* shRNAs are 29-nucleotide sequences designed to effectively knock down gene expression, while the control vector contains a scrambled shRNA sequence that does not target any specific gene. For the transfection, 1 μg of plasmid DNA was introduced into 1.5 x 10^5^ cells in 6-well plates using the ViaFect Transfection Reagent (Promega), following the manufacturer’s instructions. After transfection, a selection process with media containing puromycin was conducted to enrich for cells that were stably transfected. Total RNA was isolated from puromycin-resistant cells, followed by cDNA synthesis. Semi-quantitative PCR analyses were then performed as previously described to assess gene expression levels.

#### Wound healing assay

Once shRNA transfected cells reached close to confluency, a sterile 1000 μL pipette tip was used to create a straight line vertically along the center of each well. Each well was then gently washed twice with PBS to remove the detached cells, before replacing with fresh cell culture medium. The initial scratch (time zero) was documented using a microscope at 4X magnification. Over the following days, images of the same area in each well were captured consistently to ensure accurate tracking of the results. The gap area and its closure relative to initial scratch over time were analyzed using ImageJ.

#### Western blot analyses

Protein was extracted from whole-cell and tissue lysates, and the concentration was determined using the standard Bradford protein assay (Bio-Rad). Subsequently, a total of 10 μg of protein was loaded onto SDS-PAGE gels and post-electrophoresis, transferred onto a PVDF membrane. The membrane was blocked in 5% BSA for 1 h and incubated overnight with primary antibodies at 4°C. The next step involved an hour-long incubation with secondary antibodies at room temperature. Both prior to and following secondary antibody incubation, the membrane underwent three 5-min wash cycles with 1X TBST. Detection was facilitated by the Clarity Western ECL Substrate (Bio-Rad). The dilution ratios for the primary antibodies were as follows: actin beta (ACTB) (Cell Signaling Technologies, CST) at 1:15,000; ANKRD17 (abcam) at 1:2000; DDR1 (CST) at 1:2000; phospho-AKT (Ser473) (CST) at 1:5000; total AKT (CST) at 1:5000; STAT3 (abcam) at 1:1000 and phospho-STAT3 (abcam) at 1:1000. Secondary anti-mouse or anti-rabbit antibodies (CST) were diluted in 1% BSA at 1:2000 concentration. ImageJ was used for semi-quantitative analyses of protein bands, with the intensity of the bands was measured as an arbitrary value in relation to the expression level of ACTB.

#### RNA-sequencing (RNA-seq)

RNA sequencing and bioinformatic analyses were conducted by the Beijing Genomics Institute. The gene expression level of each gene was calculated via Illumina sequencing and expressed as fragments per kilobase of exon per million reads mapped (FPKM). Differentially expressed genes (DEGs) were identified based on gene expression level irregularities between the groups. Fold-change in gene expression between 2 samples was calculated by log2 FPKM ratio of 2 samples (Data S1). A total of 259 DEGs were identified based on the following criteria – *p* ≤ 0.05; log_2_ fold Change ≥1 (for upregulation) or ≤ −1 (for downregulation); false discovery rate (FDR) ≤ 0.05 (Data S2).

#### Immunohistochemical (IHC) staining

Tissue section slides were dewaxed by xylene (Leica) and rehydrated through a gradual decrease in ethanol concentration. Antigen epitope retrieval with Antigen Unmasking Solution (Vector Laboratories), removal of endogenous peroxidases with 3% hydrogen peroxide and blocking using M.O.M. Blocking Reagent (Vector Laboratories) for 1 h were performed on the tissue sections, followed by overnight incubation of primary antibody at 4°C in a humidified chamber. The sections were washed with PBS before incubation with horseradish peroxidase-secondary antibody for 1 h. After PBS washing, the sections were treated with freshly prepared DAB substrate (Thermo Fisher Scientific) and allowed for adequate signal development before terminating the reaction with water. The dilution ratios for the primary antibodies were as follows: ANKRD17 (Novus Biologicals) at 1:200 and phospho-DDR1 (Tyr513) (Thermo Fisher Scientific) at 1:200.

### Quantification and statistical analysis

#### Statistical analyses

Data is presented as mean ± standard deviation (SD). The assessment of statistical significance was carried out by an unpaired two-tailed Student’s *t*-test using Prism. A '*p*' value of under 0.05 was considered as indicative of statistical significance. The statistical details of these experiments can be found in the Figure legends.

## Supplemental information

Document S1. Figures S1–S6 and Table S1–S4

Data S1. RNA-seq data of control vs ANKRD17-overexpressing cells

Data S2. Differentially expressed genes (DEGs) identified based on gene expression level irregularities between control vs ANKRD17-overexpressing cells
