# Supplementary material for: ANKRD17 induces pro-survival signaling pathways that enhance cellular invasion and migration during hepatocellular carcinoma tumorigenesis
Source: iScience. 2025 Apr 17;28(5):112463. doi: 10.1016/j.isci.2025.112463 (PMC12127597; doi:10.1016/j.isci.2025.112463)
Supplement: Document S1. Figures S1–S6 and Table S1–S4 [file mmc1.pdf]

iScience, Volume *28*

Supplemental information

ANKRD17 induces pro-survival signaling pathways that enhance cellular invasion and migration during hepatocellular carcinoma tumorigenesis

Vincent W. Keng, Shan Su, Elyse S.T. Chui, Jeffrey C. To, Yao-jun Zhang, and Xiao-Xiao Li

Supplementary Table 1 Top ten enriched pathways from upregulated DEGs in *ANKRD17*-overexpressing cells

**Ingenuity Canonical Pathways**	**-log (*P*-value)**	**Ratio**	**z-score**
Cardiac hypertrophy signaling (enhanced)	1.08	0.44	3.889
STAT3 pathway	1.45	0.5	3.3
CREB signaling in neurons	0	0.241	3.207
Tumor microenvironment pathway	0.684	0.426	3.13
Systemic lupus erythematosus in B cell signaling pathway	1.41	0.481	2.746
Acute phase response signaling	1.06	0.46	2.683
Role of pattern recognition receptors in recognition of bacteria and viruses	0	0.286	2.646
Superpathway of melatonin degradation	0	0.259	2.646
IL-6 signaling	0.254	0.361	2.496
Prolactin signaling	0.693	0.5	2.449

Supplementary Table 2 Top ten enriched pathways from downregulated DEGs in *ANKRD17*-overexpressing cells

Ingenuity Canonical Pathways	-log (*P*-value)	Ratio	z-score
HIPPO signaling	2.2	0.5	2
PTEN signaling	0	0.125	2
Endocannabinoid cancer inhibition pathway	1.39	0.267	1.414
Cell cycle: G1/S checkpoint regulation	1.16	0.294	1.342
p53 signaling	0.536	0.182	1.342
AMPK signaling	0.577	0.188	1
PPARα/RXRα activation	0	0.0952	1
Role of CHK proteins in cell cycle checkpoint control	2.45	0.556	0
Cell cycle: G2/M DNA damage checkpoint regulation	2.2	0.5	0
Huntington's disease signaling	0.821	0.222	0

Supplementary Table 3 Patient data with metastasis from pathologically confirmed HCC

Patient number	Sex	Age	Metastasis site
1	M	41	Dorsal segmental nodule in the lower lobe of the right lung
2	F	62	Nodule in the lower lobe of the left lung
3	M	38	Multiple intrapulmonary metastases
4	M	70	Nodules in the apical posterior segment of the superior lobe of the left lung and in the dorsal segment of the lower lobe of the right lung
5	F	60	Lower lobe nodule of the left lung
6	M	26	Several nodules in the anterior segment of the upper lobe of the right lung and on the upper lobe of the left lung
7	M	63	Tumor metastasis in the middle right lobe of the lung
8	F	42	Hepatic tumor metastasis in the upper right lobe of the lung
9	M	40	Anterior segment of the left upper lung

All patients were of Han Chinese ethnicity

Supplementary Table 4 Primer sets used for qPCR analyses

Gene primer sets		Sequence (from 5' to 3')
ANKRD17	Forward	GTTTTATTGGCAATGCACGC
	Reverse	CAAGCTAATGTAAGGGCACTC
CTNNB1	Forward	AAAGCGGCTGTTAGTCACTGG
	Reverse	CGAGTCATTGCATACTGTCCAT
Cdh2	Forward	AGCACATGCAGTGGACATCA
	Reverse	GGGCTGATCTTTGTCCGTGA
Vim	Forward	GCAGTATGAAAGCGTGGCTG
	Reverse	GCTCCAGGGACTCGTTAGTG
Snail1	Forward	ACTACCTAGGTCGCTCTGGC
	Reverse	TGCAGCTCGCTATAGTTGGG
Mmp2	Forward	ACAAGTGGTCCGCGTAAAGT
	Reverse	AAACAAGGCTTCATGGGGGC
Vcam1	Forward	CTGGGAAGCTGGAACGAAGT
	Reverse	GCCAAACACTTGACCGTGAC
Yap1	Forward	GCCATGCTTTCGCAACTGAA
	Reverse	TCCGTATTGCCTGCCGAAAT
DDR1	Forward	ATGCCGAGGCTGACATTGTT
	Reverse	TTGGTGGCATCTGGCCGTAAG
Ddr1	Forward	CAACAGCCAGTGACGTTTGG
	Reverse	GTGGCCTGGACAAGTAGACC
IL6R	Forward	GACTGTGCACTTGCTGGTGGAT
	Reverse	ACTTCCTCACCAAGAGCACAGC
STAT3	Forward	CTTTGAGACCGAGGTGTATCACC
	Reverse	GGTCAGCATGTTGTACCACAGG
ACTB*	Forward	AGAGCTACGAGCTGCCTGAC
	Reverse	AGCACTGTGTTGGCGTACAG

* for both mouse liver and human liver cell line samples

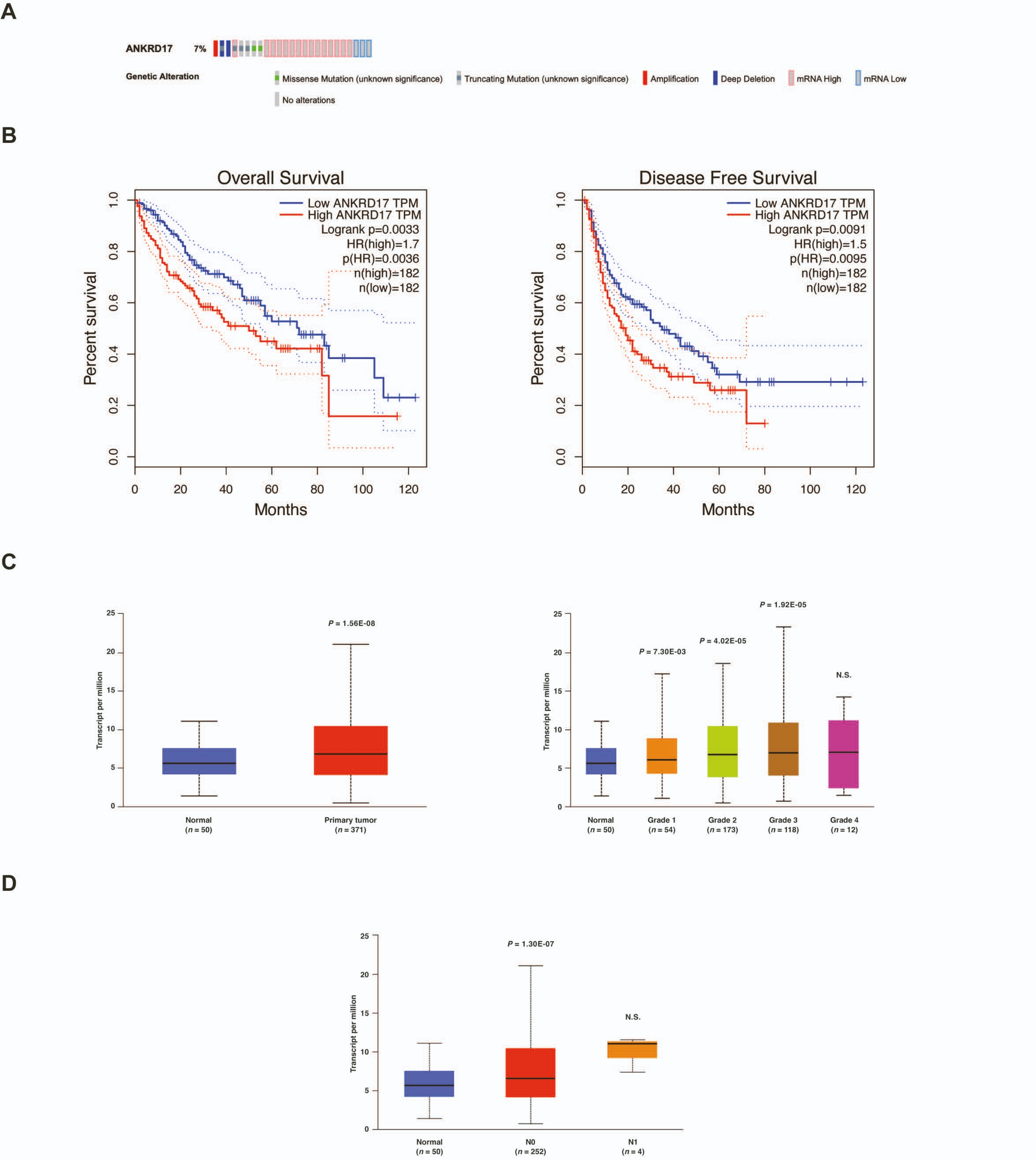

Supplementary Figure 1 Genetic alterations of *ANKRD17* in HCC patient samples. (**A**) Genetic alterations of *ANKRD17* taken from the TCGA human HCC database and visualized using OncoPrinter from cBioPortal for cancer genomics. (**B**) Statistically significant overall (**left**) and disease free (**right**) survival differences between low and high *ANKRD17* expression levels using GEPIA. Both cutoff-high and -low, 50%. (**C**) Increasing *ANKRD17* expression correlates with sample type (**left**) and tumor grade (**right**) using UALCAN. Grade 1, well differentiated (low grade); Grade 2, moderately differentiated (intermediate grade); Grade 3, poorly differentiated (high grade); Grade 4, undifferentiated (high grade). (**D**) Significantly higher *ANKRD17* expression levels have been detected in N1 HCC patients (metastases detected in 1 to 3 axillary lymph nodes) when compared to N0 HCC patients ($P = 3.63E-02$). Statistical comparisons with normal levels as shown in the graphs. N.S., non-significant.

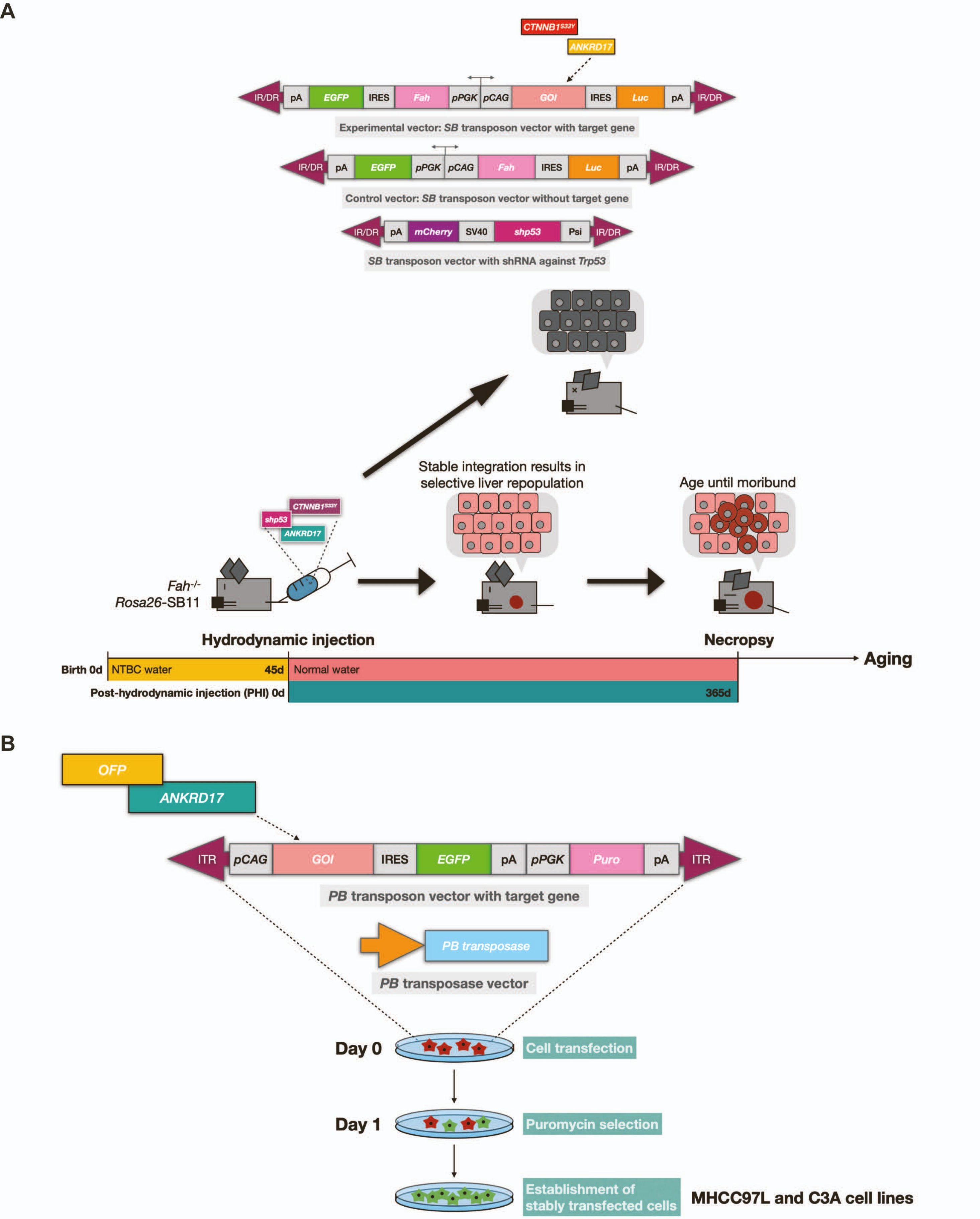

Supplementary Figure 2 *In vivo* and *in vitro ANKRD17*-overexpression studies. (**A**) Hydrodynamic tail vein injection was performed on *Fah*/SB11 mice at 45-days old to deliver *SB* transposon vectors with or without gene-of-interest (GOI) and/or *shp53* into mouse hepatocytes. NTBC supplemented water was immediately replaced with normal drinking water post-injection to allow for selective liver repopulation as a result of stable integration of transposon with *Fah* cDNA into the hepatic genome. Mice were sacrificed at around 365-days post hydrodynamic injection (PHI). (**B**) The piggyBac (*PB*) transposon system carrying GOI and *PB* transposase vectors were co-transfected into HCC cell lines (MHCC97L and C3A) at day-0. Puromycin selection medium was introduced at day-1 to enrich for positively transfected cells (green). Established stably transfected cells were then used for different downstream analyses.

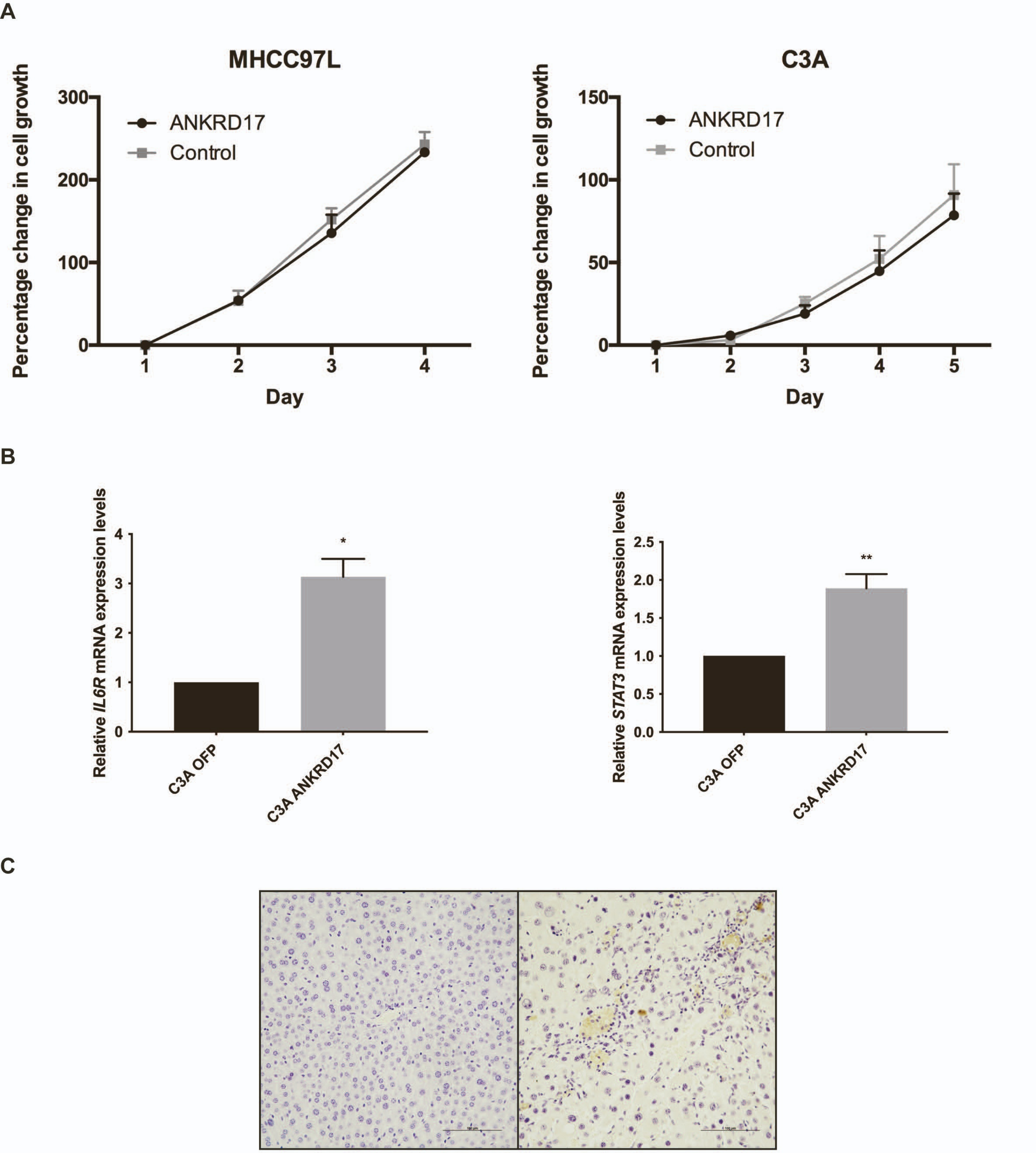

Supplementary Figure 3 *ANKRD17*-overexpression assays in human HCC cell lines. (**A**) *ANKRD17* overexpression does not induce cell proliferative rates. No significant differences were observed in the proliferative rate between *ANKRD17*-overexpressing cells compared with control orange fluorescent protein (*OFP*)-overexpressing cells for both MHCC97L (**left**) and C3A (**right**) cell lines. Data shown, mean ± S.D. (**B**) *ANKRD17*-overexpression induces *IL6* and *STAT3* expression in C3A cells when compared with control *OFP*-overexpression vector. Semi-quantitative analyses of *IL6R* (**left**) and *STAT3* (**right**), relative to *ACTB* levels, respectively, expressed as mean ± SD; *P*, unpaired Student's *t*-test: *, $P < 0.05$; **, $P < 0.01$. (**C**) Representative DDR1 IHC staining of *ANKRD17*-overexpressing (**right**) compared with normal wild-type (**left**) mouse livers. Scale bars, 100 µm.

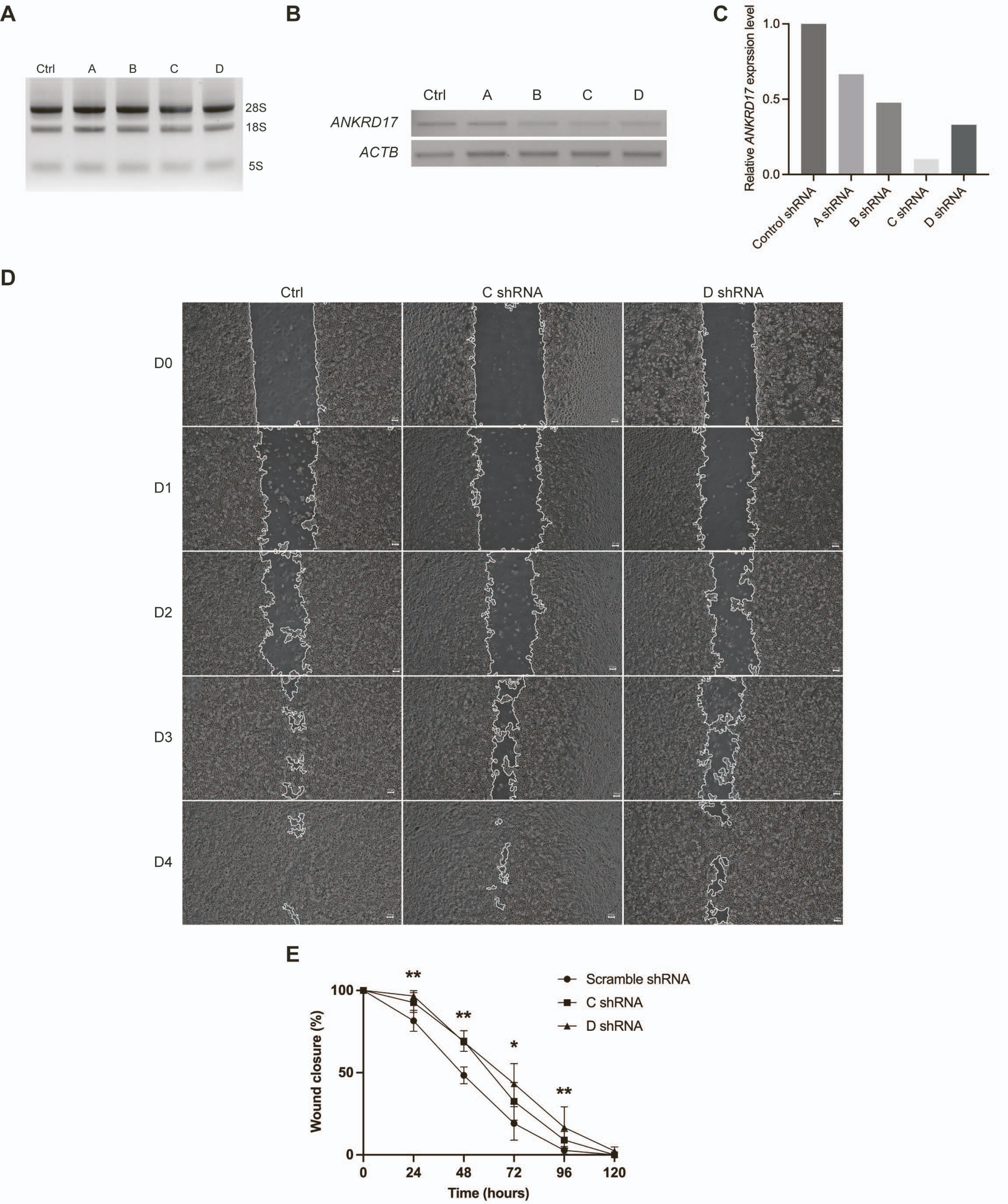

Supplementary Figure 4 RNA interference assay targeting *ANKRD17* was performed to confirm its metastatic role. (**A**) Agarose gel showing intact total RNA isolated from SNU449 cells transfected with scramble control (Ctrl) and *ANKRD17* short-hairpin RNAs (shRNAs) (A to D shRNAs). Ribosomal bands 28S, 18S and 5S. (**B**) Successful *ANKRD17* knockdown was detected in puromycin-resistant cells transfected with gene-specific shRNAs when compared with scramble control. No bands were detected in reverse transcription-negative PCR samples (*data not shown*). (**C**) Representative mean semi-quantitative analyses of *ANKRD17* expression relative to *ACTB* and normalized to scramble control in *ANKRD17* knockdown clones. (**D**) Representative images of wound healing assay performed on *ANKRD17*-knockdown clones (C and D). Areas highlighted with white lines indicate unoccupied area. D, day; scale bars, 100 μm. (**E**) Area of wound closure was calculated using ImageJ for scramble control (Ctrl) and *ANKRD17*-knockdown clones (C and D). Data shown, mean ± S.D.; **, $P < 0.05$ for both clones; *, $P < 0.05$ for D only; P, unpaired Student's t-test.

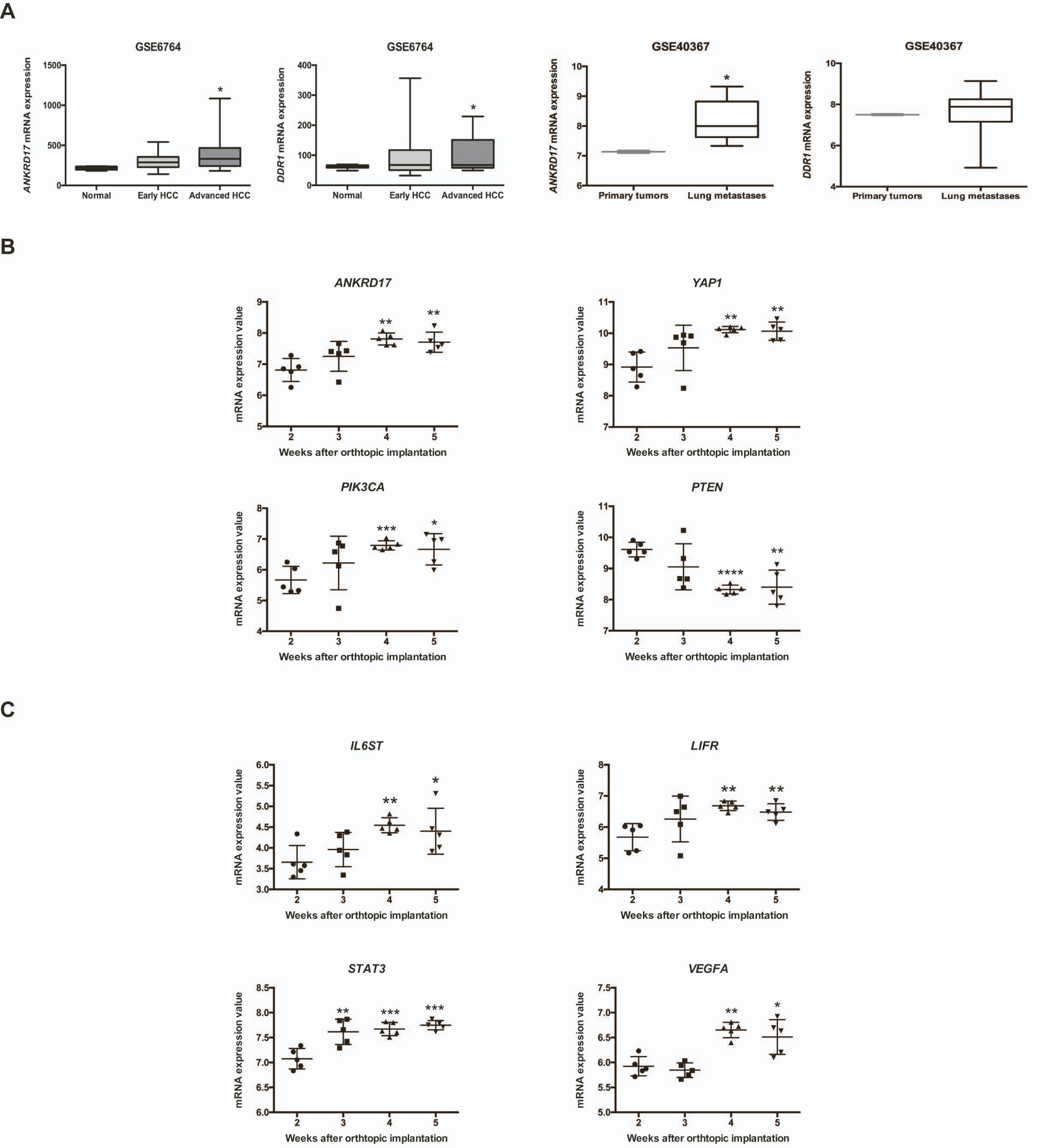

Supplementary Figure 5 Role of *ANKRD17* in HCC patients. (**A**) Expression profiling by array analyses showing increased *ANKRD17* and *DDR1* expression levels are associated with advanced stage HCC patients and metastases. *ANKRD17* and *DDR1* expression in different stages of HCC patients taken from online clinical GEO DataSets GSE6764 and GSE40367. Mean ± SD; *P*, unpaired Student's *t*-test: *, $P < 0.05$. (**B**) Expression levels of affected signaling pathways genes in orthotopically implanted HCC patient samples (GSE94016). Expression levels of *ANKRD17*, *YAP1*, *PIK3CA* and *PTEN* in orthotopically implanted HCC patient samples. (**C**) Expression levels of *IL6ST*, *LIFR*, *STAT3* and *VEGFA* in orthotopically implanted HCC patient samples.

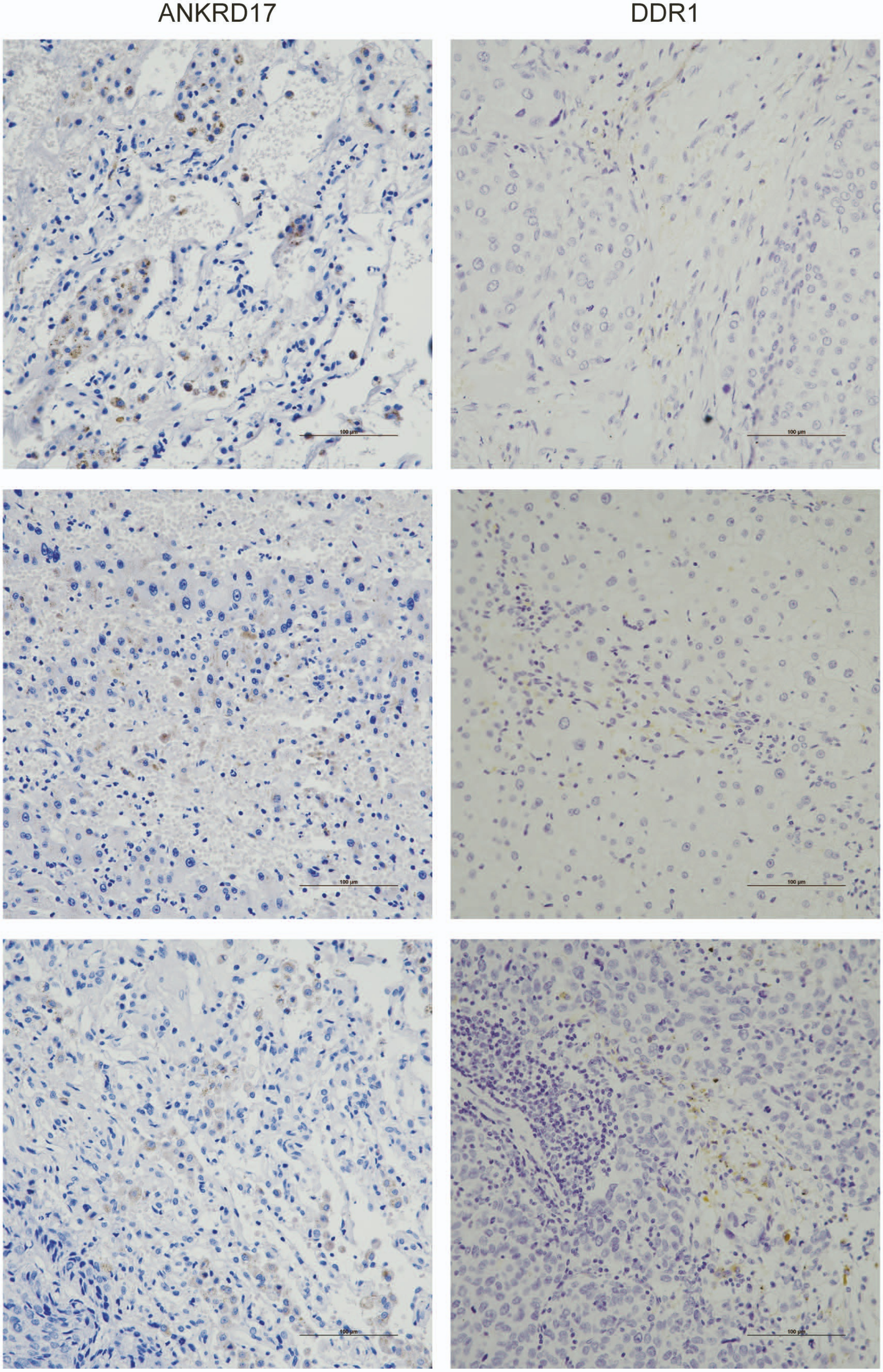

Supplementary Figure 6 Expression of *ANKRD17* and *DDR1* detected in patients with HCC-associated metastasis. Representative IHC staining of metastasis samples taken from HCC patients showing expression of both ANKRD1 (**left**) and DDR1 (**right**). Scale bars, 100 µm.
